# Genetic investigation confirmed the clinical phenotype of congenital chloride diarrhea in a Hungarian patient: a case report

**DOI:** 10.1186/s12887-019-1390-1

**Published:** 2019-01-11

**Authors:** Éva Dávid, Dóra Török, Katalin Farkas, Nikoletta Nagy, Emese Horváth, Zsuzsanna Kiss, György Oroszlán, Márta Balogh, Márta Széll

**Affiliations:** 1 Department of Pediatrics, Markusovszky Teaching Hospital, Szombathely, Hungary; 2 Department of Medical Genetics, University of Szeged, Szeged, Hungary; 3 Genetic Counseling Outpatient Clinic, Markusovszky Teaching Hospital, Szombathely, Hungary; 4 MTA-SZTE Dermatological Research Group, University of Szeged, Szeged, Hungary

**Keywords:** Congenital chloride diarrhea, *SLC26A3* gene, Compound heterozygous state, Novel mutation, Recurrent mutation

## Abstract

**Background:**

Congenital chloride diarrhea (CCD, OMIM 214700) is a rare autosomal recessively inherited condition characterized by watery diarrhea, hypochloremia and metabolic alkalosis. Mutations of the *solute carrier family 26, member 3* (*SLC26A3*, OMIM 126650) gene are responsible for the disease. The gene encodes a transmembrane protein, which is essential for intestinal chloride absorption.

**Case presentation:**

Here we report a Hungarian boy, presenting the clinical phenotype of CCD. The patient born at 32 weeks of gestation and underwent surgery for abdominal distension and intestinal obstruction related to malrotation. After recovery, electrolyte replacement therapy was necessary due to several periods of diarrhea. After exclusion of other possible causes, increased chloride concentration in the feces supported the diagnosis of CCD. The diagnosis was confirmed by molecular genetic testing. Direct sequencing revealed compound-heterozygosity for a frameshift mutation c.1295delT (p.Leu432Argfs*11) and the known Polish founder mutation c.2024_2026dupTCA (p.Ile675_Arg676insLeu).

**Conclusions:**

Here we present the clinical symptoms of the first patient in Hungary diagnosed with CCD. Based on the clinical symptoms, stool analysis and genetic testing, the diagnosis of CCD was established. Our study provides expansion for the mutation spectrum of the *SLC26A3* gene and the genetic background of CCD.

## Background

Congenital chloride diarrhea (CCD, OMIM 214700) is a rare congenital diarrhea of autosomal recessive inheritance. The leading symptoms include intrauterine onset of watery diarrhea, hypokalemia, hypochloremia, hyponatremia and metabolic alkalosis. The disorder was first described in two simultaneous case reports in 1945 [1, 2]. More than 250 cases have been reported since then. The incidence of CCD is variable worldwide. Most cases are sporadic; however, CCD occurs more frequently in Finland, Poland, and in countries around the Persian Gulf, especially in Kuwait and in Saudi Arabia [3, 4]. The gene for CCD, *solute carrier family 26 member 3* (*SLC26A3*, NM_000111) is located on chromosome 7q22.3-q31.1 [3, 5] and encodes a transmembrane protein, which is an apical epithelial Cl^−^/HCO_3_^−^ exchanger expressed on the surface epithelium of the ileum and colon. The malfunction of this anion transporter results in deficient absorption of Cl^−^ and secretion of HCO_3_^−^. So far, more than 70 mutations of the gene – including the founder mutations from Finland, Poland and Arabic countries – are known to be pathogenic (http://www.hgmd.cf.ac.uk). In the present paper, we report the first discovered case of CCD in Hungary and its genetic investigation.

## Case presentation

**Fig. 1:**
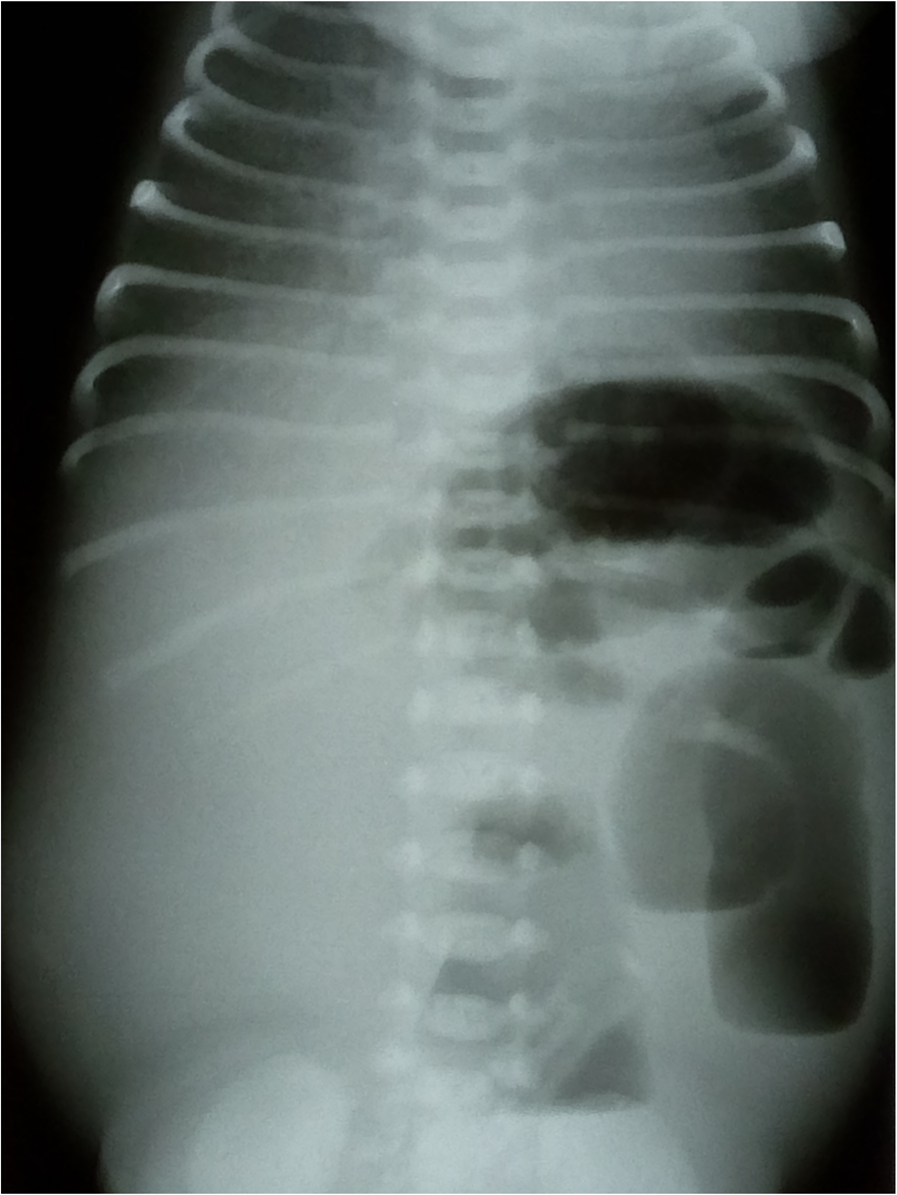
Simple abdominal radiography. Imaging study on the first life day shows small bowel ileus

The premature boy, born by Cesarean section at 32 weeks of gestation, had a birthweight of 1850 g and Apgar scores of 6/1 and 9/5. Prenatal ultrasound was performed at 24 weeks of gestation and showed small bowel dilatation of the fetus and increased flow in the ascending aorta. The newborn had abdominal distension and had thin, fluid-like discharge from the rectum. Abdominal radiographs showed small bowel ileus (Fig. 1). On postnatal day 2, laparotomy was performed revealing a type 1 malrotation of the gut associated with obstructing Ladd’s Bands. On postnatal day 12, the newborn developed symptoms of late-onset sepsis caused by *Klebsiella pneumoniae*. Gastrointestinal bleeding, pulmonary hemorrhage and a small intracranial hemorrhage were also present. The patient had bilious emesis and failure to pass stool. Imaging studies showed dilated loops of the bowel; therefore, detorquation of a bowel loop and adhesiolysis were performed. After reoperation, he tolerated feeding and gained weight; however, repeated blood analyses indicated hyponatremia, hypochloremia, hypokalemia and metabolic alkalosis, necessitating prolonged oral electrolyte replacement therapy. Sweat test showed normal chloride concentrations and direct sequencing of the coding regions of the *cystic fibrosis transmembrane conductance regulator* (*CFTR*) gene revealed a single delta F508 mutation. The infant had frequent loose stools, sometimes 6–10 times per day. In the first year of life, he was frequently admitted to the hospital because of viral infections and dehydration, and needed electrolyte replacement in increasing amounts. A 24-h urine collection test showed low concentrations of sodium, potassium and chloride ions (9.7 mmol/l, 14.9 mmol/l and 6.6 mmol/l, respectively). Plasma renin activity (34.2 mg/ml/h) and aldosterone (51 mg/dl) levels were increased, suggesting secondary hyperaldosteronism. After exclusion of other possible causes, chloride loss in the feces was detected. Elevated chloride concentrations were detected in centrifuged feces samples (148 and 154 mmol/l; normal range is below 90 mmol/l) using standard chemistry analysis. Based on the high chloride levels in the stool, diagnosis of CCD was established.

**Fig. 2:**
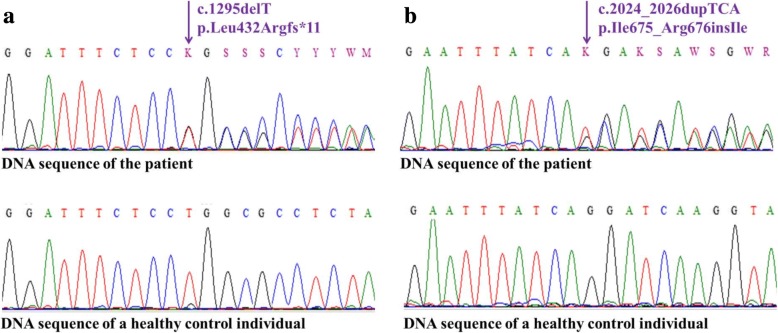
Identification of two mutations of the *SLC26A3* gene. Direct sequencing revealed (**a**) a deletion of a T base (c.1295delT, p.Leu432Argfs*11) in exon 11 and (**b**) a duplication of TCA bases (c.2024_2026dupTCA, p.Ile675_Arg676insIle) in exon 18 of the gene. Both mutations were present in the affected patient in heterozygous state

**Fig. 3:**
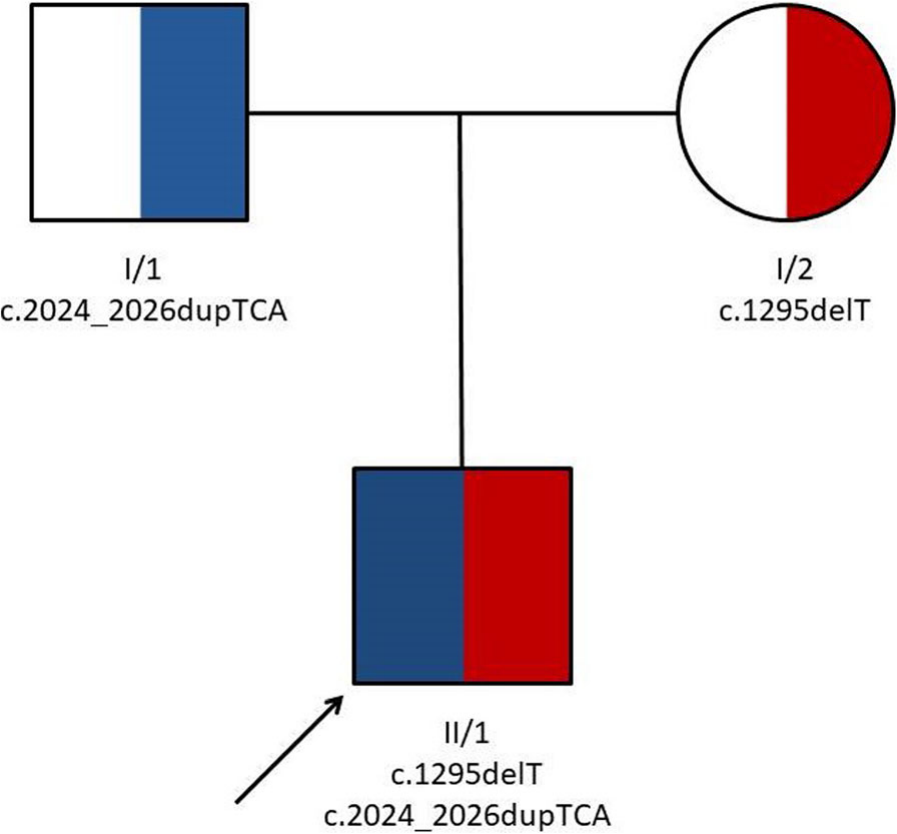
Pedigree of the investigated Hungarian patient. The patient (II/1) was the only clinically affected individual in his pedigree: his parents (I/1 and I/2) were clinically unaffected

To perform molecular genetic confirmation, peripheral blood samples were drawn from the affected patient and from his unaffected family members (*n* = 2), as well as from unrelated healthy controls (*n* = 50), and genomic DNA was isolated from the blood samples. The coding regions and the flanking introns of the *SLC26A3* gene were amplified and sequenced. Direct sequencing of the investigated regions of the *SLC26A3* gene revealed two heterozygous mutations: a novel thymine-base deletion (c.1295delT, p.Leu432Argfs*11, Fig. 2a) in exon 11 and a recurrent 3-base (TCA) duplication (c.2024_2026dupTCA, p.Ile675_Arg676insIle, Fig. 2b) in exon 18. After the disease-causing mutations were identified in the patient, the mutation status of the parents was determined (Fig. 3). The clinically unaffected parents carry the mutations in heterozygous form, and all unrelated healthy controls (*n* = 50) carry the wild type sequence.

After establishing the diagnosis, oral electrolyte supplementation was continued with 2,1 g of NaCl and 2,2 g of KCl a day, and proton pump-inhibitor therapy was also administered to inhibit gastric Cl^−^ secretion. [6, 7] Since then no parenteral fluid replacement therapy was necessary, as electrolytes were in normal range and alkalosis did not recurred. 12 months after the conclusion of the genetic analysis, the patient has stools still 6–8 times a day, but successfully underwent toilet training. Currently at the age of 4, his weight is at 25 percentile (16 kg), his psychomotor development is appropriate for age, he attends nursery school.

## Discussion and conclusions

CCD is a rare autosomal recessive disorder caused by homozygous and compound heterozygous mutations of the *SLC26A3* gene. The clinical diagnosis is mainly based on the clinical history, characteristic metabolic abnormalities and high levels of chloride in stool. The symptoms and signs frequently overlap with other conditions, such as cystic fibrosis and Bartter syndrome. Clinical history as well as urinary and sweat chloride levels help in the differentiation of these diseases (Table 1) [8–10].

**Table 1:** Comparison of the clinical phenotype of differential diagnostic disorders

Disorder	CCD	Bartter syndrome	Cystic fibrosis
Characteristic			
Onset of disease	Presents early in neonates	Presents in infants, but can present later	Presents primarily in neonates and infants
Cl^−^ concentration in urine	Low	High	Low
Cl^−^ concentration in stool	Elevated in watery stool	Not detected in normal stool	Not detected in fatty or normal stool
Sweat Cl^−^ test results	Can be elevated	Normal	High
Blood pH	Metabolic alkalosis	Metabolic alkalosis	Metabolic alkalosis
Possible electrolyte disturbances	Hyponatremia, hypochloremia and hypokalemia	Hyponatremia, hypochloremia and hypokalemia	Hyponatremia, hypochloremia and hypokalemia

Due to absence of known hotspots in the *SLC26A3* gene in the Hungarian population, molecular genetic confirmation of the CCD clinical diagnosis is usually performed by sequencing the entire coding region of the gene. Most of the known mutations are point mutations or small deletions, many of which affect exon 3–6 and 12–15 [10]. In populations in which founder effects have been observed (Finland, Poland, Saudi Arabia and Kuwait), single-mutation analysis of the typical founder mutation is the first test performed [4].

The present case is the first Hungarian patient diagnosed with CCD. The condition in the index patient was caused by compound heterozygous mutations in the *SLC26A3* gene. A novel thymine-base deletion (c.1295delT, p.Leu432Argfs*11) was identified in exon 11 (Fig. 3a) leading to the development of a premature termination codon. This newly identified mutation has not been reported in any SNP database (ExAC, 1000 Genome Project, ESP). The pathogenicity of this novel variation was suggested by the clinical context and the fact that the mutation truncates the 3′ half of the predicted protein. A recurrent 3-base (TCA) duplication (c.2024_2026dupTCA, p.Ile675_Arg676insIle) was detected in exon 18 (Fig. 3b), resulting in the incorporation of an additional amino acid into the protein. This recurrent disease-causing mutation is a founder mutation in the Polish population and is involved in almost 50% of the CCD-associated cases in Poland [4]. Parents of the reported patient are not aware of any Polish relatives.

Undiagnosed CCD is a severe condition. Only rare cases are known to have survived after the first year undiagnosed. In an affected child, an acute gastroenteritis can lead to life-threatening dehydration and hypokalemia. Lifelong electrolyte supplementation to correct the biochemical abnormalities with NaCl and KCl is the basis of management. Timely and adequate therapy determines the outcome, normal growth and development of the affected child and requires early and sufficient supplementation. Patients require fastidious follow-up to prevent long-term complications, such as renal failure and hyperuricemia [11]. A correct diagnosis of CCD has an important impact on family planning and allows preimplantation genetic diagnosis and prenatal diagnostics.

## Abbreviations

CCD: Congenital chloride diarrhea
CFTR: Cystic fibrosis transmembrane conductance regulator
SLC26A3: Solute carrier family 26 member 3
